# Turgor loss point explains climate‐driven growth reductions in trees in Central Europe

**DOI:** 10.1111/plb.13687

**Published:** 2024-06-28

**Authors:** N. Kunert, I. K. Münchinger, P. Hajek

**Affiliations:** ^1^ Functional and Tropical Plant Ecology University of Bayreuth Bayreuth Germany; ^2^ Department of Integrative Biology and Biodiversity Research Institute of Botany, University of Natural Resources and Life Sciences Vienna Austria; ^3^ Geobotany, Faculty of Biology University of Freiburg Freiburg im Breisgau Germany

**Keywords:** Drought, growth anomalies, leaf drought tolerance traits, temperate forest

## Abstract

- As climate change thrives, and the frequency of intense droughts is affecting many forested regions, a mechanistic understanding of the factors conferring drought tolerance in trees is increasingly important. However, studies linking the observed growth reduction to mechanistic traits are still rare.
- We compared the median growth anomalies of 16 native tree species, gathered across a network of study plots in Bavaria, with the mean species‐specific turgor loss point (π_tlp_) measured at five locations in Central Europe
- π_tlp_ explained 37% of the growth anomalies observed in response to the intense droughts between 2018 and 2020 compared to the pre‐drought period between 2006 and 2017 across sites.
- π_tlp_ constitutes an important leaf drought tolerance trait and influences the growth response of native tree species during extraordinary dry periods. As climate change‐induced droughts intensify, tree species with drought‐tolerant leaves will be less vulnerable to growth reductions. π_tlp_ provides a useful indicator for selecting tree species to adapt forest management systems to climate change.

## Graphical abstract

Leaf turgor loss point of native tree species shows a high predictive power over the observed growth anomalies in Central Europe during the extensive drought period between 2018 and 2020.

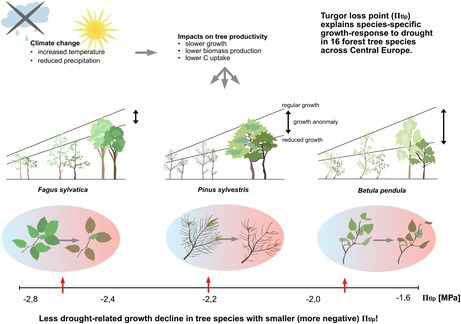

## INTRODUCTION

Climate change has emerged as one of the most pressing global challenges of our time, with far‐reaching consequences for our planet's forest ecosystems. One of the most alarming manifestations of climate change is the intensification of droughts and heavily altered precipitation patterns (Trenberth *et al*. 2014; Dai *et al*. 2018; IPCC 2021). Intense droughts are posing a significant threat to forest productivity (Hofhansl *et al*. 2015; Meakem *et al*. 2018) and are causing accelerated forest die‐off, as can be observed across terrestrial biomes worldwide (Allen *et al*. 2010; Cobb *et al*. 2017; Senf *et al*. 2020). Despite numerous observational and manipulative studies intending to quantify net changes in forest productivity in response to drought, the future remains unknown. Due to the vast diversity of tree species worldwide the predictions of changes in forest productivity will remain challenging. Therefore, trait based approaches have shown high potential to cluster species into functional groups and have allowed some generalizations on how forest ecosystems might change. This, in turn, facilitates the selection of suitable tree species for upcoming climate‐resilient forest management systems, with a specific emphasis on properties such as drought tolerance.

Despite possible osmotic adjustment of leaves in response to drought (e.g., Hesse *et al*. 2023), numerous studies have shown that the leaf turgor loss point (π_tlp_), i.e., the leaf water potential at which the leaf loses turgor and wilts permanently, is a strong predictor in explaining current species distribution as a response to historical water availability. For example, there are clear patterns with regard to linkages of π_tlp_ with local topographic water availability (Maréchaux *et al*. 2015; Bartlett *et al*. 2016; McFadden *et al*. 2019), across rainfall gradients (Baltzer *et al*. 2008; Blackman *et al*. 2019; Medeiros *et al*. 2019; Kunert *et al*. 2021), and spanning biomes (Bartlett *et al*. 2012; Bartlett, Klein *et al*. 2016; Bartlett, Zhang *et al*. 2016; Zhu *et al*. 2018; Vargas *et al*. 2022). The strong correlation of π_tlp_ with species distribution in response to water availability across environmental variability indicates that π_tlp_ is a measure of a plant's capacity to maintain cell turgor during times of water limitation (Zhu *et al*. 2018). With declining turgor pressure stomata close, stomatal conductance declines, and gas exchange, including the uptake of CO_2_, ceases. There is increasing evidence that π_tlp_ predicts the leaf water potential at which 50% of stomatal conductance is lost (Brodribb *et al*. 2003; Blackman *et al*. 2010; Bartlett, Zhang *et al*. 2016). Species with a more negative π_tlp_ also have higher stomatal conductance under limited soil water availability. Hence, species characterized by a more negative π_tlp_ should be able to take up and assimilate carbon under drier conditions than species with a less negative π_tlp_, which is, in turn, essential for plants to maintain growth under increasingly dry conditions. However, studies directly linking π_tlp_ to drought responses in plant growth are still rare. For example, McGregor *et al*. (2021) demonstrated that π_tlp_, among other traits, explained growth responses during and after exceptionally dry years in an unmanaged species‐rich deciduous temperate forest. In this particular forest, temperate tree species with a less negative π_tlp_ were more likely to experience growth declines in summer drought years, but the study only described these responses at very limited spatial scales. It remains unclear whether π_tlp_ represents an appropriate trait to predict tree growth responses to drought also across large spatial scales.

Here we present, for the first time, evidence for the efficacy of π_tlp_ as a predictor of drought response in relation to the recorded growth declines in Central European tree species and across a variety of forest management systems. We identify π_tlp_ as an important driver of drought resistance, by integrating the effects of drought on tree growth documented in Thom *et al*. (2023).

## MATERIAL AND METHODS

### Leaf turgor loss point and tree growth data

In this study, we combined two different datasets – one on leaf turgor loss point and another on drought‐induced tree growth anomalies – to examine the short‐ and long‐term responses of tree growth to drought. The dataset on leaf turgor loss point (π_tlp_) was collected for 16 native species at five different locations distributed across Central Europe, representing considerable variation in mean annual temperature (MAT) and precipitation (MAP) (Table 1). The π_tlp_ data were collected in the rural district of Fürth in northern Bavaria, Germany; the Traunstein ForestGEO plot (Davies *et al*. 2021) located in south‐eastern Bavaria, Germany; the Vienna Woods north‐west of Vienna, Austria; the IDENT field site in Freiburg in south‐western Germany (Tobner *et al*. 2014); and Bayreuth in northern Bavaria, Germany. All sites represent managed forests, with the exception of IDENT‐Freiburg, which is an experimental tree plantation (cf. Tobner *et al*. 2014). The forests located in Bavaria are dominated by coniferous tree species, such as Scots pine (*Pinus sylvestris* L.) and/or Norway spruce (*Picea abies* L.), with broadleaved species such as pedunculate oak (*Quercus robur* L.) and European beech (*Fagus sylvatica* L.), but the broadleaved species are less abundant within these forests (Giammarchi *et al*. 2017; Kunert 2020). However, the forest in Austria is dominated by European beech accompanied by a variety of different oak species (Kunert & Hajek 2022). These five locations span a climatic gradient wiht regard to MAT and MAP. MAT ranges from 8.9 to 11.6 °C, with Bayreuth being the coldest and Freiburg being the warmest location. MAP ranges between locations from 740 to 1060 mm, with Vienna being the driest location and Traunstein the wettest of the five locations.

**Table 1:** Sampling locations where π_tlp_ was measured and location characteristics.

				Fürth	Traunstein	Vienna	Freiburg	Bayreuth
			Location	49.410000°N, 10.827583°E	47.935000°N, 12.666400°E	48.228693°N, 16.249912°E	48.019444°N, 7.826944°E	49.9261188°N, 11.5841229°E
			Altitude	320 m a.s.l.	590 m a.s.l.	180 m a.s.l.	240 m a.s.l.	340 m a.s.l.
			MAP	830 mm	1060 mm	740 mm	880 mm	960 mm
			MAT	9.7 °C	9.1 °C	10.9 °C	11.6 °C	8.9 °C
Common name	Latin name	Family	Species code	π_tlp_ available	π_tlp_ available	π_tlp_ available	π_tlp_ available	π_tlp_ available
Silver fir	*Abies alba*	Pinaceae	ABIAL	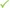	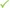	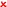	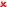	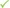
Field maple	*Acer campestre*	Sapindaceae	ACECA	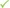	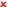	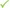	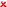	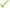
Sycamore	*Acer pseudoplatanus*	Sapindaceae	ACEPS	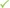	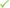	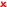	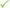	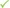
Common alder	*Alnus glutinosa*	Betulaceae	ALNGL	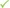	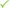	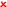	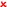	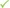
Silver birch	*Betula pendula*	Betulaceae	BETPE	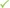	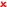	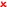	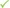	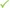
European hornbeam	*Carpinus betulus*	Betulaceae	CAPBE	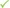	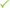	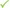	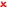	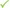
European beech	*Fagus sylvatica*	Fagaceae	FAGSY	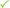	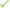	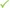	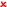	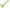
European larch	*Larix decidua*	Pinaceae	LARDE	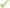	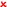	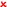	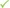	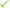
Norway spruce	*Picea abies*	Pinaceae	PICAB	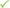	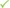	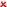	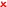	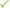
Scots pine	*Pinus sylvestris*	Pinaceae	PINSY	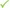	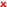	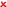	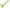	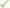
Common aspen	*Populus tremula*	Salicaceae	POPTR	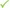	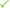	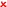	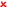	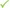
Wild cherry	*Prunus avium*	Rosaceae	PRUAV	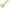	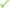	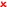	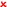	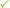
Sessile oak	*Quercus petraea*	Fagaceae	QUEPA	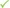	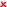	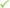	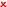	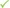
Pedunculate oak	*Quercus robur*	Fagaceae	QUERO	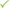	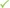	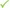	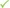	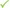
Small‐leaved lime	*Tilia cordata*	Malvaceae	TILCO	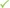	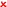	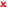	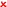	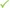
Wych elm	*Ulmus glabra*	Ulmaceae	ULMGL	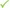	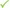	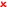	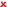	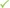
			Source	Kunert & Tomaskova (2020)	Münchinger *et al* (2023)	Kunert & Hajek (2022)	unpublished	unpublished
			Study period	7–18 July 2019	22 June 2022	28–29 June 2021	18–26 June 2021	26–30 June 2023

Leaf turgor loss point of native tree species has high predictive power for the observed growth anomalies in Central Europe during the extensive drought period between 2018 and 2020.

The dataset on tree growth (basal area increment) was taken from Thom *et al*. (2023). Basal area increment was collected on 200 plots scattered across 15 ecoregions in Bavaria, Germany. The 15 ecoregions range from the Alps in the south to the lowlands in the northwest, representing a gradient of different climate and edaphic conditions. The forests in Bavaria mainly contain Norway spruce, Scots pine, European beech, and pedunculate oak, along with other tree species. The historic variation in MAT and MAP across geographic ecoregions was 3.3–9.6 °C, and 579–2660 mm, respectively.

**Fig. 1:**
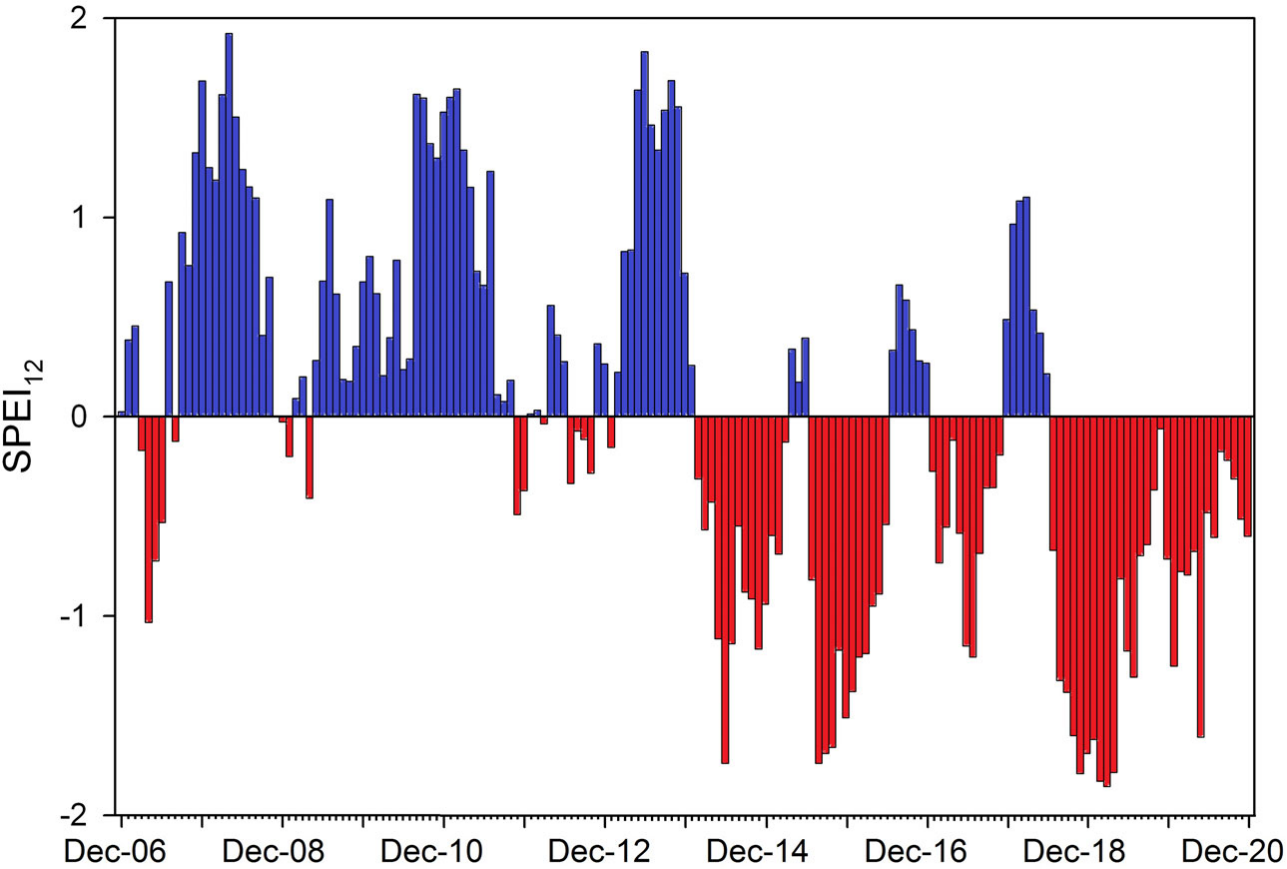
Average drought conditions across Bavaria for the years 2007–2020. Bars represent monthly SPEI, accounting for the conditions of the previous 12 months (SPEI12). Monthly mean temperature and monthly precipitation for the period 2006–2020 were provided by the German Meteorological Service (DWD, retrieved from https://opendata.dwd.de). Drought period was defined by Thom *et al*. (2023) as the years 2018–2020.

Thom *et al*. (2023) defined the years 2018–2020 as a period of intense drought, also noting those om previous years, particularly 2014 and 2015, were comparatively hot and dry, as indicated by negative Standardized Precipitation Evapotranspiration Index (SPEI; Fig. 1). Therefore, water stress cannot be entirely excluded during the reference period (2006–2017), but the average temperature was 1.8 °C lower and precipitation 130 mm higher compared to the drought period throughout these years (2018–2020). Furthermore, the SPEI was also negative during the winter months of the drought period, whereas in the reference period the SPEI was mostly positive during winter (Fig. 1). Those 3 years of consecutive dry growth periods without full soil water re‐storage during the winter months has been broadly accepted to have caused widespread issues with water availability across many regions in Europe (e.g., van der Wiel *et al*. 2023).

### Cross‐location estimation of πtlp

The water potential at turgor loss point (π_tlp_) was assessed in all five climate regions following the protocol described in Kunert (2020). Therefore, leaf osmotic potential at full hydration (π_osm_) was measured with a vapour pressure osmometer (VAPRO 5520; Wescor, Logan, UT, USA) following the rapid assessment method of Bartlett, Scoffoni, & Sack (2012). At least three tree individuals of each species were sampled per location. One sun‐exposed branch was collected from each tree individual during the growing season when leaves were fully expanded. Sampled branches were brought to the laboratory immediately after cutting. Branches were placed in humid and opaque plastic bags to prevent dehydration during transport. All branches were recut underwater in the laboratory at least two nodes distal to the original cut, placed in partly water‐filled buckets, and covered with opaque plastic bags. We applied the standing rehydration method to avoid over‐rehydration during sample preparation (Arndt *et al*. 2015). After rehydrating overnight, two leaves were sampled per branch and individual. Per leaf, one disc was cut out with a 4‐mm diameter cork borer. The disc was wrapped in aluminium foil, submerged in liquid nitrogen (LN_2_), and perforated after shock‐freezing using a dissection needle. The standard 10 μl chamber well of the osmometer (VAPRO 5520; Wescor) was used and the osmometer was set to the auto‐repeat mode. All osmometer readings were recorded until equilibrium was indicated between readings (difference between readings <0.01 MPa). The solute concentration value c_0_ (in mmol·kg^−1^) was converted into the osmotic potential at full hydration (π_osm_) using the following equation:

(1) $$\pi_{\mathrm{osm}}=\left(R\times T\right)/1000\;c_{0}$$

where R is the ideal gas constant, and T, the temperature in degrees Kelvin. Leaf water potential at turgor loss (π_tlp_) was estimated from the equation established byBartlett, Scoffoni, & Sack (2012):

(2) $$\pi_{\mathrm{tlp}}=0.832\;{\pi_{\mathrm{oms}}}^{-0.631}$$

### Estimation of growth anomalies

Species‐specific tree growth anomalies were estimated following Thom *et al*. (2023). Briefly, Thom *et al*. (2023) used data from a permanent inventory network across Bavaria. The network serves to assess vitality of the forested area at regular intervals. Inventory plots are located on a regular 16 × 16 km raster grid (in some areas at 8 × 8 km raster grid). Each plot is 150 × 150 m in size, and at each corner an angle‐count sampling is conducted accounting for all trees with a diameter at breast height (DBH) > 7 cm. To estimate the relative growth anomalies, the average basal area increment between 2006 and 2017 was used as the reference growth point. The relative growth anomalies were calculated as (Drought/Reference−1) × 100. Since the data on annual growth anomalies were not normally distributed and the record counts for certain species were limited, median and interquartile range estimates of relative growth changes per species were derived for the years 2018 to 2020, corresponding to growth alterations over three consecutive years: 2017 to 2018, 2018 to 2019, and 2019 to 2020. In addition, significance testing was conducted to determine whether the medians of annual relative growth anomalies significantly differed from 0, representing the growth rate during the reference period of 2006 to 2017. This evaluation employed a nonparametric sample test (Helwig 2019), which is close to the approach of the Wilcoxon‐Signed‐Rank Test, with permutations included to increase resistance against smaller sample sizes (Thom *et al*. 2023).

### Statistical analysis

In this study, we focused on 16 Central European tree species showing significant negative growth anomalies in response to drought (Table 1). To evaluate the bivariate relationship between the mean π_tlp_ of tree species sampled at five climate regions in Central Europe and the relative growth anomalies reported by Thom *et al*. (2023), Pearson's product–moment correlation coefficient was employed and was tested for significance by comparing *t*‐values. Assumptions of normality and homogeneity of variance were tested using Shapiro–Wilk and Levene's test, respectively. We used a linear mixed effects model to test how much variance can be explained by random effects (R package ‘lmerTest’, Kuznetsova *et al*. 2015). We used relative growth anomalies as dependent variable, π_tlp_ and species as fixed effects, and location as random factor. We tested not only species means (n = 52), but also individual means of all measured tree individuals (n = 171). Standardized Precipitation Evapotranspiration Index (SPEI) was compiled using the ‘spei’ package (Vicente‐Serrano *et al*. 2010). The data analysis was conducted using the R program, version 4.3.1 (R Core Team 2023).

## RESULTS

**Fig. 2:**
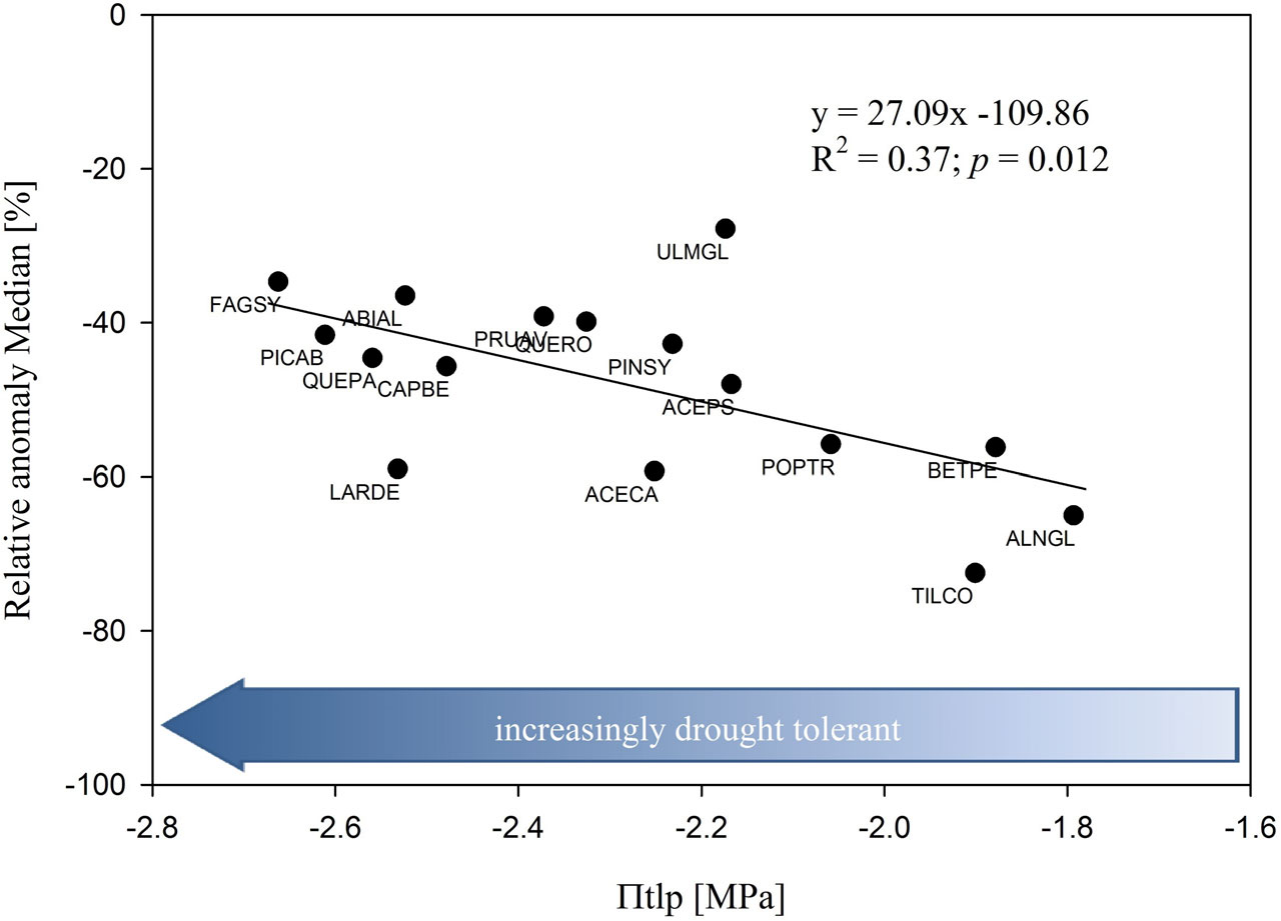
Relationship between mean π_tlp_ of 16 native tree species, sampled at five different locations in Central Europe (see Table 1) and medians of relative growth anomalies observed during the drought period 2018–2020 in Bavaria. Blue arrow indicates the direction of increasing drought tolerance with increasingly more negative π_tlp_.

Relative growth anomalies correlated negatively with mean π_tlp_ (y = −27.09x−109.86; *R* ^2^ = 0.37; *P* = 0.012) and therefore species with more negative mean π_tlp_ showed a lower growth reduction in response to drought than species with a less negative mean π_tlp_ (Fig. 2). For example, *Fagus sylvatica*, with the lowest cross‐location mean π_tlp_ of −2.66 ± 0.09 MPa showed an accordingly low relative growth reduction of −34.7%, whereas *Tilia cordata*, the species with a less negative π_tlp_ of −1.90 ± 0.21 MPa had the highest growth reduction of 72.5%. We had one outlier, *Ulmus glabra*, showing the lowest growth reduction of −27.8% with a π_tlp_ of −2.17 ± 0.20 MPa. Overall, mean π_tlp_ explained 37.0% of the variation in growth response among the investigated species. Location explained 0.67% of the variance of the relationship at species level and 1.07%, considering individual means at tree level.

## DISCUSSION

In this study, we found strong evidence that species mean turgor loss point (π_tlp_) as a leaf‐level drought tolerance trait largely explains the observed growth anomalies of a specific tree species in response to the intense droughts between 2018 and 2020. Growth anomalies observed across tree species in response to drought were negatively correlated with species‐specific π_tlp_. Hence, tree species with a more negative π_tlp_ were characterized by a lower reduction in growth after the intensive drought in the final years. This is the first study, to our knowledge, linking π_tlp_ to growth reduction during drought across large spatial scales, spanning a variety of ecoregions, and therefore covering regional climatic differences. This supports the premise that the observed growth anomalies were driven by fundamental physiological mechanisms linked to leaf‐level drought resistance, represented by π_tlp_. Our findings underline the importance of π_tlp_ as a ‘higher‐level’ trait in regard to drought resistance. Despite possible limitations in the predictive power of physiological properties measured at individual and/or detached organs (e.g., Larcher 2001), π_tlp_ has been recognized to reflect whole plant water relationships (Bartlett, Scoffoni, Ardy *et al*. 2012; Bartlett, Scoffoni, & Sack 2012). π_tlp_ is, therefore, a very direct metric of plant drought tolerance. A more negative π_tlp_ means that plants remain turgid and maintain function because of their ability to function with more negative leaf water potentials (Sack *et al*. 2003; Lenz *et al*. 2006), i.e., ability to sustain tree growth performance even under water limitations. Besides the linkage of π_tlp_ to the individual plant hydraulic function, π_tlp_ has been associated with habitat filtering on local, regional and pan‐regional scales in natural forest ecosystems (Medeiros *et al*. 2019; Kunert *et al*. 2021; Vargas *et al*. 2022). Studies linking π_tlp_ to direct measures of stress responses under drought, such as growth reductions, are still limited in number (see McGregor *et al*. 2021). Therefore, our study is of great importance as it identifies π_tlp_ as a trait influencing tree growth responses of a variety of species in response to drought and across large spatial scales. In a broader sense, tree growth rates are a conception explanatory variable for tree mortality (Harmon & Bell 2020), and reduced growth rates during drought could potentially indicate an emerging trend in a specific tree species leading to accelerated tree mortality in response to drought (Sapes *et al*. 2019). As π_tlp_ partly explains growth anomalies, it also might have great potential to indicate a species‐specific risk for hydraulic failure under drought stress and describe the probability that a certain tree species will die under intensive drought stress (compare Álvarez‐Cansino *et al*. 2022). However, the two economically important species currently undergoing massive large‐scale die‐off events in Central Europe and beyond are the two conifers, namely European spruce (*Picea abies*) and Scots pine (*Pinus sylvestris*). These two species showed moderate growth anomalies of −41.6% and −42.8%, respectively. This highlights the limitations of predicting tree mortality via growth modelling approaches. Both of these species have differing hydraulic characteristics at leaf level, with European spruce (mean regional π_tlp_: −2.61 ± 0.21 MPa) being a rather drought tolerant species, and Scots pine (mean regional π_tlp_: −2.23 ± 0.02 MPa) being a drought sensitive species. This is further evidence that there are diverging causes of tree mortality. High mortality rates might be more related to thermal sensitivity than drought stress, particularly for European spruce (Kunert 2020). Overall, the actual cause of death might involve a set of mutualistic mechanisms (Hajek *et al*. 2022).

In conclusion, this study advances our knowledge of factors explaining the susceptibility of tree species to drought and provides confirmative evidence that π_tlp_ has considerable predictive power for tree species performance under a changing climate. Furthermore, π_tlp_ is an easily measurable trait, in contrast to other hydraulic traits, such as stem‐level traits, in particular P50 or P88, which are time‐consuming to measure. Therefore, π_tlp_ might provide an important tool for pre‐assessing the suitability of a tree species for establishment in climate‐affected forests. To further confirm π_tlp_ as an important drought tolerance trait, a research priority should be to test for seasonal stability of π_tlp_ in response to changes in environmental conditions and site differences. This could be achieved by implementing common garden experiments controlling for species adaptations, and consideration of site‐specific factors that might modulate growth responses to drought.

## Author contributions

NK and PH designed the study. IKM, PH and NK collected the data. NK and IKM performed the statistical analysis. NK wrote the first version of the manuscript. All authors contributed to drafting the final version of the manuscript.

## Conflict of interest

The authors declare no conflict of interest.
